# Niraparib enhances antitumor immunity and contributes to the efficacy of PD-L1 blockade in cervical cancer

**DOI:** 10.1007/s00432-024-05819-x

**Published:** 2024-06-13

**Authors:** Jie Chang, Shimin Quan, Sijuan Tian, Shirui Wang, Simin Li, Yanping Guo, Ting Yang, Xiaofeng Yang

**Affiliations:** Department of Gynecology and Obstetrics, The First Affiliated Hospital of Xi’an Jiaotong University, Xi’an, Shaanxi China

**Keywords:** Cervical cancer, Niraparib, PD-L1 blockade, PARP inhibitor, ICB

## Abstract

**Purpose:**

With the development of immunotherapy research, the role of immune checkpoint blockade (ICB) in the treatment of cervical cancer has been emphasized, but many patients still can’t receive long-term benefits from ICB. Poly ADP ribose polymerase inhibitor (PARPi) has been proved to exert significant antitumor effects in multiple solid tumors. Whether cervical cancer patients obtain better benefits from the treatment regimen of PARPi combined with ICB remains unclear.

**Methods:**

The alteration of PD-L1 expression induced by niraparib in cervical cancer cells and its underlying mechanism were assessed by western blot and immunofluorescence and quantitative real-time polymerase chain reaction (qRT–PCR).The regulation of PTEN by KDM5A was confirmed using Chromatin immunoprecipitation (ChIP) assay and RNA interference. Analyzing the relationship between PD-L1 and immune effector molecules through searching online databases. Therapeutic efficacy of niraparib, PD-L1 blockade or combination was assessed in syngeneic tumor model. The changes of immune cells and cytokines in vivo was detected by immunohistochemistry (IHC) and qRT–PCR.

**Results:**

We found that niraparib upregulated PD-L1 expression and potentiated the antitumor effects of PD-L1 blockade in a murine cervical cancer model. Niraparib inhibited the *Pten* expression by increasing the abundance of KDM5A, which expanded PD-L1 abundance through activating the PI3K-AKT-S6K1 pathway. PD-L1 was positively correlated with immune effector molecules including TNF-α, IFN-γ, granzyme A and granzyme B based on biological information analysis. Niraparib increased the infiltration of CD8^+^ T cells and the level of IFN-γ, granzyme B in vivo.

**Conclusion:**

Our findings demonstrates the regulation of niraparib on local immune microenvironment of cervical cancer, and provides theoretical basis for supporting the combination of PARPi and PD-L1 blockade as a potential treatment for cervical cancer.

## Introduction

Cervical cancer (CC) is a globally prevalent life-threatening disease, with approximately 600,000 new cases reported annually (Sung et al. 2021), and advanced cervical cancer remains poorly prognosis because of the increased probability of local metastasis and distant recurrence (Liontos et al. 2019). The therapeutic regimen of cisplatin and paclitaxel combined with bevacizumab is currently considered as frontline treatment (Marquina et al. 2018), but the improvement in prognosis is still poor.

Immunotherapy are promising across different cancer, and ICB agents have been approved for the treatment of patients with advanced cancer. Currently, the use of pembrolizumab as first-line chemotherapy for recurrent cervical cancer is confined to patients with PD-L1 positivity (Combined Positive Score, CPS≥1), but the therapeutic results have not been satisfactory. In KEYNOTE-028, the objective response rate (ORR) of patients with advanced cervical cancer treated with pembrolizumab was only 17% (Frenel et al. 2017). In CheckMate 358 clinical study, the ORR of patients with recurrent/metastatic HPV-associated cervical cancer after treatment with pembrolizumab was 26.3% (Naumann et al. 2019). Therefore, it is necessary to explore novel combination therapy options to maximize clinical benefits.

Multiple immunosuppressive mechanisms cause resistance to anti-PD-L1/PD-1 therapy, such as the lack of cancer antigens, reduced infiltration of T cells in tumor tissue, and the production of immunosuppressive factors in the TME (Kim and Chen 2016). PARPi has been shown to exert antitumor effects in a variety of solid tumors. In addition to synthetic lethality, PARP inhibitors have been proven to regulate the TME by activating immune pathways and up-regulating the expression of PD-L1 in cancer cells, which enhances the efficacy of PD-L1/PD-1 blockade (Pantelidou et al. 2019; Kim et al. 2020; Shen et al. 2019; Peyraud and Italiano 2020). Therefore, PARPi in combination with ICB is expected to solve the dilemma of poor effect of monotherapy and provide a new strategy for cancer treatment. However, few studies have been conducted on PARPi monotherapy or combined therapy for cervical cancer. The efficacy of the combination therapy involving PARPi and ICB in cervical cancer is uncertain, and the underlying molecular mechanisms remain unclear.

In this study, we aimed to clarify the regulatory effects of niraparib on the immune microenvironment of cervical cancer and its potential molecular mechanisms. Additionally, the therapeutic efficacy of niraparib in combination with PD-L1 monoclonal antibody against cervical cancer was explored.

## Materials and methods

### Cell lines and culture conditions

SiHa and HeLa cell lines were obtained from the Center for Translational Medicine, the First Affiliated Hospital of Xi’an Jiaotong University, China. The U14 mouse cervical cancer cell line was purchased from the Fu Heng Cell Bank (Shanghai Fuheng Biology Science and Technology). All cell lines were cultured in high glucose DMEM media (Hyclone, USA) with 10% fetal bovine serum (FBS) (Biological Industries, Israel) and maintained in a humidified incubator with 5% CO_2_ at 37 °C.

### Western blotting (WB)

Cell samples were prepared as previous described (Zhang et al. 2020).Briefly, cells were lysed with RIPA Lysis Buffer containing 1 mM PIC and 1 mM PMSF on ice for 10 min, centrifuged at 13,000 rpm for 15 min, and the supernatant was collected. For membrane protein extraction, the cells were collected at 4℃ and washed twice with pre-cooled PBS. The membrane protein was extracted using a membrane protein extraction kit (Proteintech, USA) following the instructions. For the nuclear protein analysis, nuclei was isolated according to the procedure of the nuclear extraction kit (Solarbio, China). Protein was denatured by adding loading buffer (Zhonghui, China) and boiling at 100℃ for 10 min. Proteins were resolved by 10% SDS polyacrylamide gel electrophoresis and transferred onto pre-activated PVDF membrane (Millipore, USA). The membrane was blocked with 5% skimmed milk at room temperature for 1 h and incubated with specific antibodies for overnight at 4 °C. The primary antibodies used were as follows: anti-KDM5A (1:750, A4755, ABclonal, China), anti-PD-L1 (1:500, WL02778, Wanlei, China), anti-PTEN (1:750, WL01901, Wanlei, China), anti-AKT (Ser473) (1:2000, 4060, Cell Signaling Technology, USA), anti-p70s6k1 (Thr389) (1:1000, 9205, Cell Signaling Technology, USA), anti-AKT (1:2000, 9272, Cell Signaling Technology, USA), anti-p70s6k1 (1:1000, 9202, Cell Signaling Technology, USA). Subsequently, the PVDF membranes were incubated with the corresponding HRP-conjugated secondary antibodies for 1 h at room temperature. The immunoreactive bands were detected using enhanced chemiluminescence reagent (Millipore, USA) and measured with Image J Software.

### Immunofluorescence (IF) staining

The cells were inoculated in a confocal dish and subjected to specific treatments before immunofluorescence staining. For PD-L1 staining, the cells were fixed and blocked with 5% BSA, followed by incubation with primary antibodies (anti-PD-L1 (1:50, Sc-19091, SantaCruzBiotechnology, USA), anti-KDM5A (1:50, A4755, ABclonal, China)) at 4 °C overnight. For KDM5A staining, permeabilization with Triton-X100 was performed before fixation, and the remaining steps were performed as previously described. The cell samples were then stained with the relevant secondary antibodies for 1 h at room temperature in a dark room. Finally, the cell nuclei were counterstained with DAPI Fluoromount-G (SouthernBiotech, USA). Stained slides were imaged using inverted fluorescence microscope (Leica).

### Chromatin immunoprecipitation (ChIP) assay

The ChIP assay was performed in accordance with the operating instructions of the ChIP Assay Kit (Beyotime, China). In short, HeLa cells were incubated with 1% formaldehyde for 10 min at 37℃ for crosslinking, and glycine solution was added to terminate the reaction. Then, the cell sample was washed with pre-cooled PBS containing 1 mM PMSF and incubated in SDS lysis buffer on ice for 10 min, and sonicated to shear the genomic DNA. The supernatant was extracted by centrifugation at 12,000 g for 5 min at 4 °C. KDM5A antibody (2 mg/mL, Abclonal, China) and IgG antibody (2 mg/mL, Beyotime, China) were added to the sonicated lysates, mixed and shaken overnight at 4 °C. On the following day, the mixture was incubated with Protein A+G Agarose/Salmon Sperm DNA on a rotator at 4 °C for 60 min, which was successively washed for multiple times, eluted and finally de-crosslinked with 5 M NaCl. DNA enrichment was established by PCR using 2× Taq PCR StarMix with Loading Dye (GenStar, China). PTEN promoter regions were tested using DNA agarose gel electrophoresis. The promoter specific primers are listed in Table 1.

**Table 1:** Primer sequences

Name	Sequence	Application
KDM5A-sense	GACCGACAUUGGUGUAUAUTT	Gene silencing
KDM5A-antisense	AUAUACACCAAUGUCGGUCTT	Gene silencing
S6K1-628-sense	GUGGAGGAGAACUAUUUAUTT	Gene silencing
S6K1-628-antisense	AUAAAUAGUUCUCCUCCACTT	Gene silencing
Negative control-sense	UUCUCCGAACGUGUCACGUTT	Gene silencing
Negative control-antisense	ACGUGACACGUUCGGAGAATT	Gene silencing
PTEN-CHIP-F1	CCGACATTTGTCTGAACTGT	ChIP-PCR
PTEN-CHIP-R1	GAGCGCTGTCGGGGCTGGCG	ChIP-PCR
PTEN-CHIP-F2	GAAGCCCTCCGACCAGGCTC	ChIP-PCR
PTEN-CHIP-R2	GCCCGCTCCGGTGCCCCCAA	ChIP-PCR
PTEN-CHIP-F3	TGATACACGCTGGCGACACAATAG	ChIP-PCR
PTEN-CHIP-R3	CGCTGCTCAGTGTAGAGGGAAATG	ChIP-PCR
PTEN-CHIP-F4	ACGCACCCATCTCAGCTTTC	ChIP-PCR
PTEN-CHIP-R4	GAGCATGCCCAGTGTAGCTG	ChIP-PCR
PTEN-forward	ACCAGAGACAAAAAGGGAGTA	RT–qPCR
PTEN-reverse	ACCACAAACTGAGGATTGCA	RT–qPCR
KDM5A-forward	TTACCAACAGGTCAGACGCAT	RT–qPCR
KDM5A-reverse	GGTTTGCTACATTCCTCGGCG	RT–qPCR
GZMB-forward	GTGTGCTATGTGGCTGGTTGG	RT–qPCR
GZMB-reverse	ACTCCCGATCCTTCTGTACTGTC	RT–qPCR
IFNG-forward	GGAGGAACTGGCAAAAGGATGG	RT–qPCR
IFNG-reverse	CAGGTGTGATTCAATGACGCTTATG	RT–qPCR

### RNA interference

SiHa and HeLa cell lines were transfected with negative control, KDM5A or S6K1 specific small interfering RNA (siRNA) oligo (Tsingke Biotechnology, China), the following siRNA sequences for the negative control, KDM5A and S6K1 are listed in Table 1. SiHa and HeLa cells were inoculated into 6-well plates in appropriate quantities, and the transfection complex (containing Lipofectamine 2000 reagent and siRNA sequences at a ratio of 1:1) was prepared in serum-free medium for transient transfection. The medium was changed to complete medium after 6 h, and the protein or RNA was extracted for subsequent experiments after 48 h.

### Quantitative real‑time polymerase chain reaction (qRT–PCR)

Total RNA was isolated from the cells using RNAfast200 (Fastagen, China). The RNA samples were quantified using a Nano-300 micro-spectrophotometer (ALLSHENG). Total RNA (1 μg) was reverse-transcribed with HiFiScript gDNA Removal RT MasterMix (CWBIO) at 42℃ for 2 min, 37℃ for 15 min, and 85℃ for 5 s. Two-step qRT–PCR was conducted using SYBR Green II Premix (Takara) following the manufacturer’s protocol. GAPDH was used as an internal control. The cycle threshold (CT) was used as the representative point. Related gene expression levels were assessed using the following formula: ${\text{RQ}} = 2^{ - \Delta \Delta C_{\text{q}} }$. The primers used for qRT–PCR are listed in Table 1.

### Immunohistochemical (IHC) analysis

The tumor specimens were fixed in 4% paraformaldehyde and embedded in paraffin. Four millimeter sections from the paraffin blocks of tumors were used in subsequent experiments. Sections were dewaxed in dimethylbenzene, hydrated with gradient ethanol, and the antigen was repaired. The nonspecific protein binding was blocked with normal goat serum (SP-9001, Zsbio, China) at 37℃ for 1 h. Tumor sections were incubated with primary antibodies against CD8 (GB114186, Servicebio, China), CD163 (GB11340-1, Servicebio, China) and Ki67 (9449, Cell Signaling Technology, USA). The Image J software was used for signal intensity analysis.

### Data mining

The correlation between PD-L1 and the infiltration of CD8^+^ T cells as well as immune effector molecules was investigated using the data extracted from the Tumor Immune Estimation Resource (TIMER) 2.0 (http://timer.comp-genomics.org/).

### Syngeneic tumor model treatment protocol

Studies involving laboratory animals followed the ARRIVE guidelines. GemPharmatech provided 16 female 6-week-old specific pathogen-free (SPF) C57BL/6 mice (Chengdu, China). The mice were maintained in a SPF room with free access to standard laboratory animal feed and sterile water at the Experimental Animal Center of Xi’an Jiaotong University (license number SCXK (Shaanxi) 2006-001). The mice were raised in a specific environment for 7 days to acclimatize them to their surroundings prior to the experiments. U14 cancer cells (5 × 10^6^) were subcutaneously injected into the right armpit. The Mice were randomly divided into four treatment groups as follows: control group using PBS, intraperitoneally injected 4 times per week; niraparib group (50 mg/kg), intraperitoneally injected 4 times per week; PD-L1 blockade group using rabbit mAb#10F.9G2 (10 mg/kg), intraperitoneally injected twice per week; or a combination of niraparib and PD-L1 blockade treatment group, dosage and frequency as previously described. Tumor size and the weight of mice were measured and recorded every 3 days after treatment. Tumors were harvested on day 22, and the organs were extracted for the assessment of drug toxicity. The Experimental Animal Ethics Committee of Xi’an Jiaotong University authorized all operations in the study following the Animal Experimentation Ethics Guidelines (approval number: XJTUAE2023-2128).

### Statistical analysis

Statistical analyses were performed using SPSS 25.0 software. Unpaired Student’s *t* test was used for the comparison of two independent samples. One-way ANOVA supplemented with LSD-T was implemented to analyze multiple sets of data. The data are presented as mean ± SEM. Statistical significance was set at *P* < 0.05.

## Results

### Niraparib inhibits the proliferation of cervical cancer cells and enhances PD-L1 expression

**Fig. 1:**
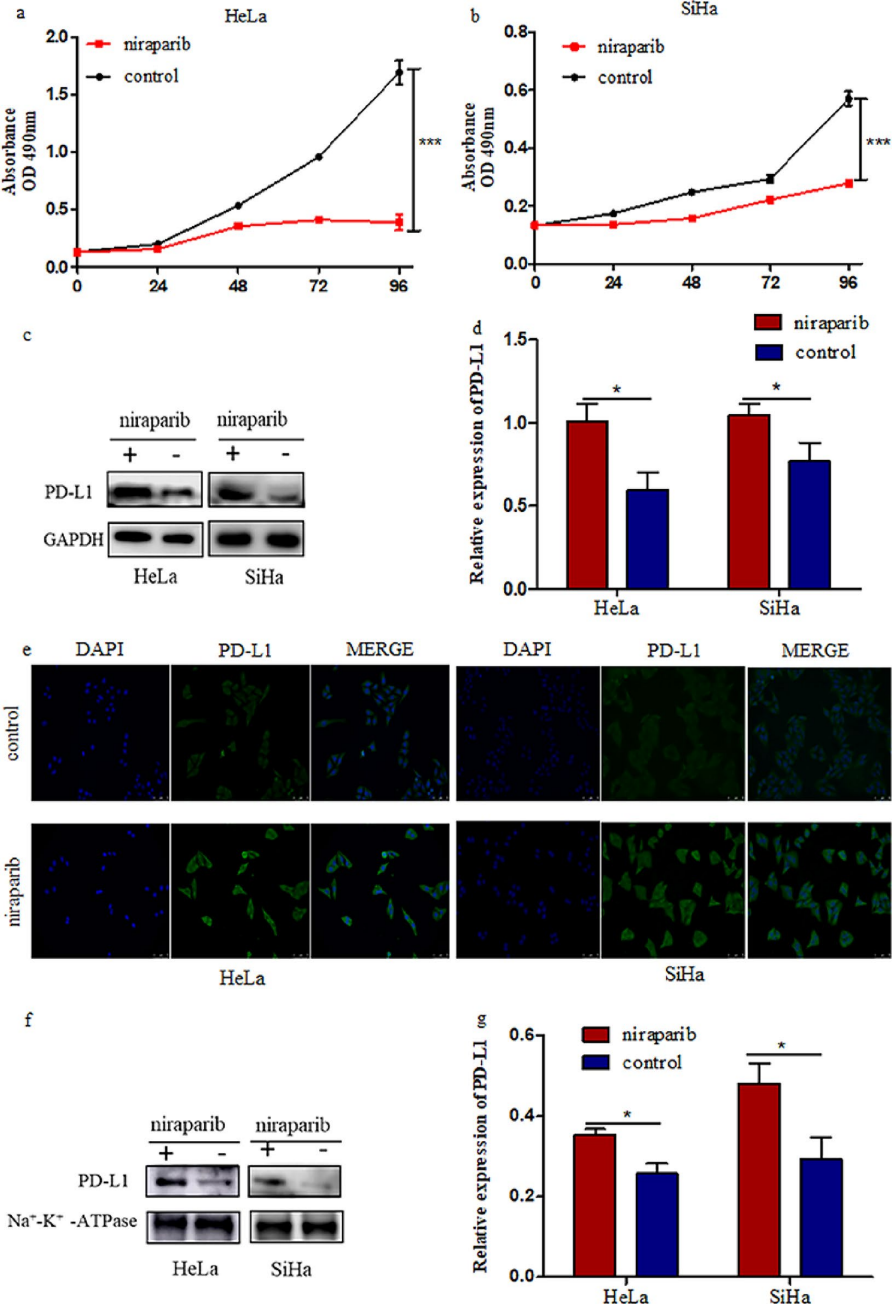
Niraparib could play antitumor and enhance PD-L1 expression in cervical cancer cells. **a**,**b** The proliferation of HeLa and SiHa under the action of niraparib was detected by MTT. **c** The protein levels of PD-L1 in HeLa, SiHa cells after treatment with niraparib detected by western blotting. **d** Densitometric analysis of PD-L1 protein levels in cervical cancer cells with different treatments. **e** Representative immunofluorescence staining of PD-L1 expression in SiHa and HeLa cells with different treatment. Magnification, ×200. **f** Western blotting analysis of PD-L1 on the membrane of cervical cancer cells under the effect of niraparib. **g** Densitometric analysis of PD-L1 protein levels on cervical cancer cells membrane with different treatments. *** *P* < 0.001,* *P* < 0.05

PARPi monotherapy or combination therapy for cervical cancer is rarely studied. To investigate the antitumor efficacy of niraparib in cervical cancer cells, we treated SiHa, HeLa cells with niraparib for 24h, 48h, 72h, 96h and analyzed cell viability by MTT assay. The results showed that niraparib significantly inhibited the proliferation of cervical cancer cells (Fig. 1a, b). Several studies have confirmed that PARP inhibitors exert anticancer activity through multiple pathways besides synergistic lethality. Our finding confirmed that niraparib could increase PD-L1 expression in cervical cancer cells (Fig. 1c–e). Next, the cell membrane proteins was extracted to investigate the changes of PD-L1 expression in cervical cancer cells. As shown in Fig. 1f and g, niraparib increased the expression of PD-L1 on the cell membrane of HeLa and SiHa cells. Together, these results indicated that niraparib can increase the total and cellular surface PD-L1 expression in cervical cancer cells.

### Niraparib activates PI3K-AKT-S6K1 signaling axis to upregulate PD-L1 expression in cervical cancer cells

**Fig. 2:**
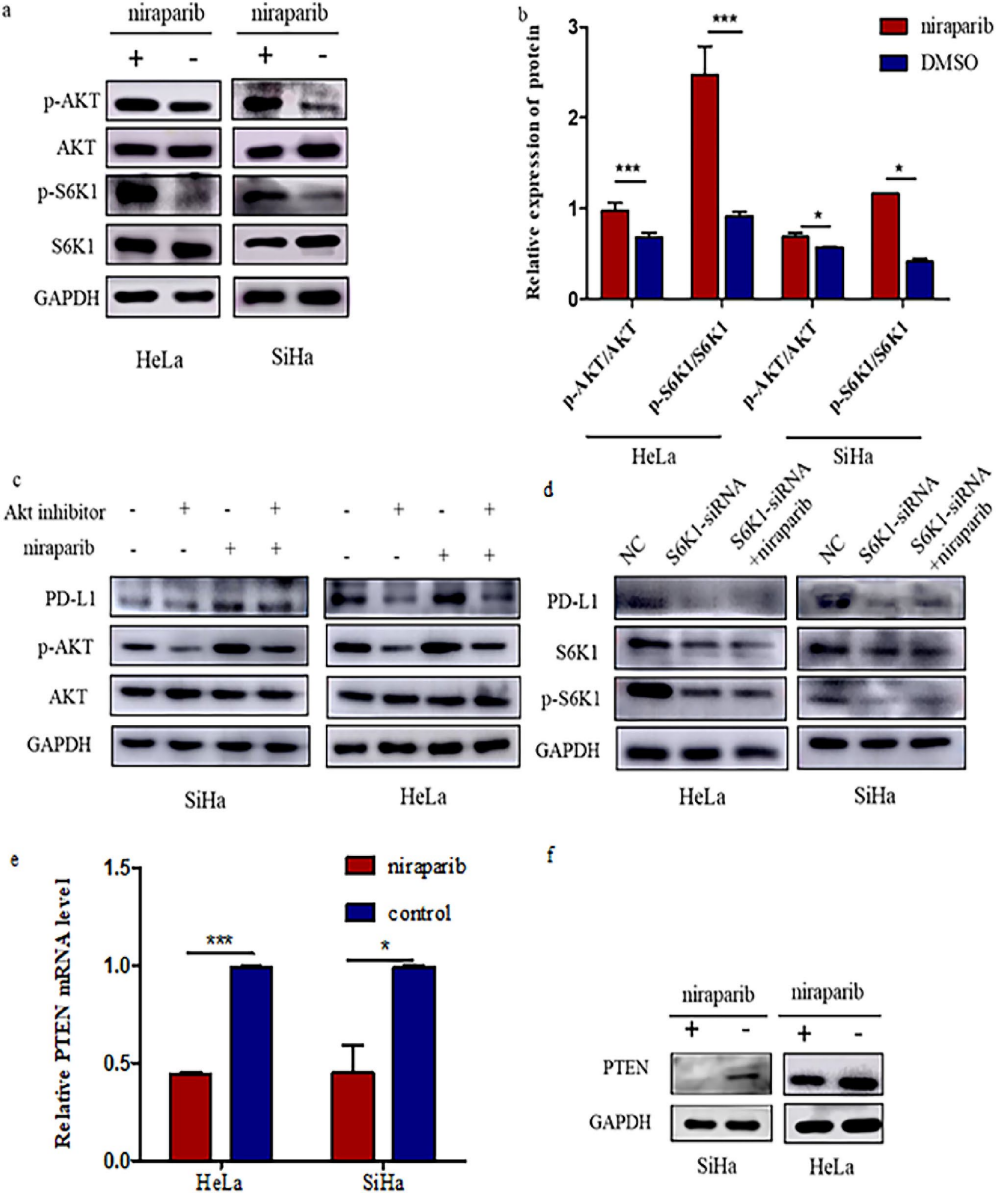
Niraparib increases PD-L1 abundance by activating PI3K-AKT-S6K1 cascade. **a**,**b** SiHa and HeLa cells in the growth index stage were selected and treated with 20 μmmol/L niraparib. The molecular alterations related to PI3K-AKT-S6K1 signaling pathway were detected by western blotting. **c** Western blotting analysis of the expression of p-AKT and PD-L1 in SiHa and HeLa cells after treatment with AKT inhibitor and niraparib. **d** Western blot analysis was performed to assess the effects of niraparib on S6K1, p-S6K1 and PD-L1 protein expression following transfection with S6K1 siRNA in SiHa and HeLa cells. **e** Relative expression of PTEN mRNA in SiHa and HeLa cells after treatment with niraparib. **f** The expression of PTEN in cervical cancer cells after treating with 20μmmol/L niraparib, analyzed by western blotting. *** *P* < 0.001,* *P* < 0.05

Previous results showed that niraparib can drive PD-L1 upregulation in cervical cancer cells, but the intrinsic molecular mechanism is not clear. Our results showed that niraparib activated the PI3K-AKT-S6K1 signaling pathway and increased the expression levels of p-AKT and p-S6K1 in cervical cancer cells (Fig. 2a, b). When the signaling pathway was blocked by the addition of an AKT inhibitor (MK2206), the expression of PD-L1 in cervical cancer cells decreased. The decrease of PD-L1 expression in cervical cancer cells exposed to the AKT inhibitor corresponded with the decreased activation of AKT, and niraparib alleviated this decrease (Fig. 2c). Transfection of SiHa and HeLa cells with S6K1 siRNA resulted in decreased expression of S6K1, p-S6K1, and PD-L1 proteins, as determined by Western blot analysis. Notably, the subsequent addition of niraparib for 24 h did not significantly alter the expression levels of p-S6K1 or PD-L1 in either SiHa or HeLa cells (Fig. 2d). Our results indicated that niraparib’s regulatory effect on PD-L1 expression was contingent upon the activation of the PI3K-AKT-S6K1 signaling pathway.

Phosphatase and tensin homolog (PTEN) can inhibit tumors through the PI3K-AKT pathway, in which PTEN negatively regulates the pathway by dephosphorylating the second growth factor messenger, PIP3, to PIP2. Next, we examined the effect of niraparib on PTEN expression in cervical cancer cells. The results showed that niraparib inhibited Pten transcription and reduced its expression (Fig. 2e, f). Collectively, these results indicated that niraparib enhanced PD-L1 expression in cervical cancer by activating the PI3K-AKT-S6K1 signaling pathway through the inhibition of PTEN.

### Niraparib enhances the expression of KDM5A and decreases *Pten* transcription to up-regulate PD-L1

**Fig. 3:**
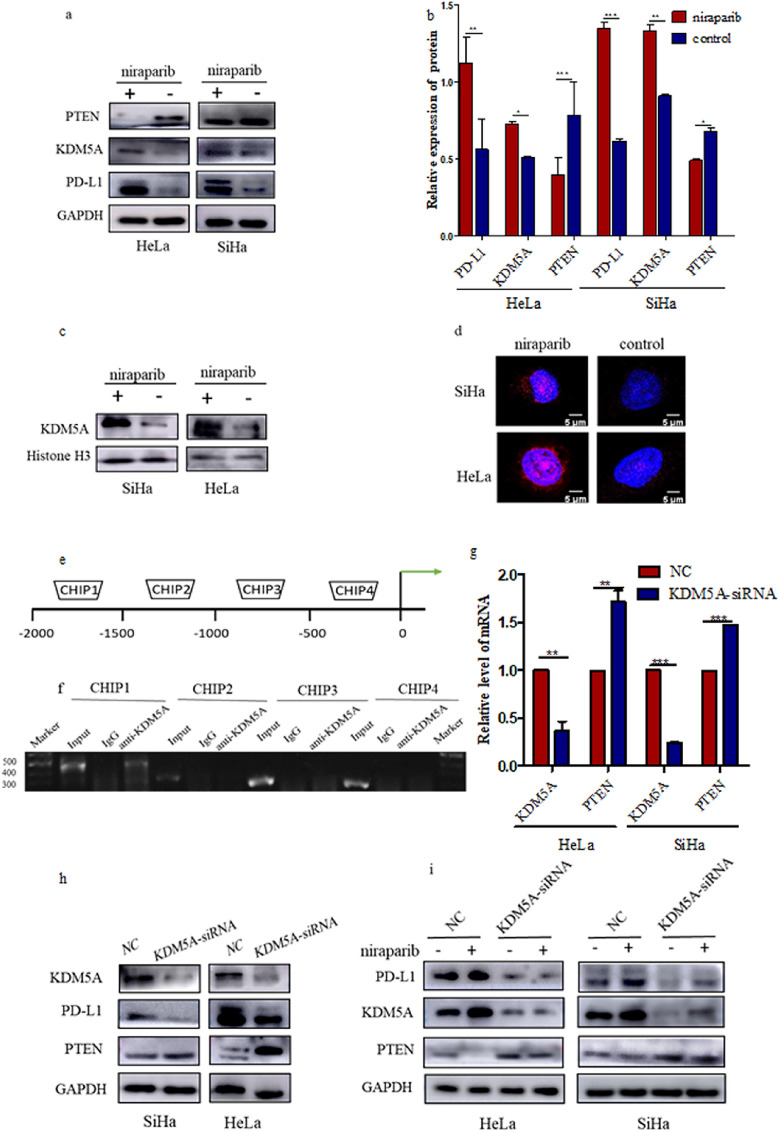
Niraparib decreases PTEN abundance and increases PD-L1 abundance through KDM5A. **a**,**b** Western blotting analysis of KDM5A, PTEN and PD-L1 abundance in Hela, Siha cells treated with niraparib or DMSO. **c** Western blotting analysis of KDM5A abundance in the nuclear fractions of HeLa, SiHa cells treated with niraparib or with DMSO as negative control (NC). **d** Representative immunofluorescence staining of KDM5A expression in SiHa and HeLa cells. **e**,**f** ChIP analysis of the *Pten* promoter in HeLa cells treated with niraparib. **g** Detection the mRNA levels of KDM5A and PTEN in SiHa and HeLa cells by RT-qPCR. **h** Western blotting analysis of NC and KDM5A-knockdown cervical cancer cells with antibodies against the indicated proteins. **i** Western blotting analysis of PTEN, KDM5A and PD-L1 in NC or KDM5A-knockdown cervical cancer cells treated with niraparib. *** *P* < 0.001, ** *P* < 0.01,* *P* < 0.05

We further explored the potential mechanism underlying the changes in PTEN expression caused by niraparib. Studies have shown that histone demethylase KDM5A activates PI3K-AKT-S6K1 signaling by inhibiting PTEN, which affects the response rate to anti-PD-L1 immunotherapy (Wang et al. 2020). Moreover, KDM5A interacts with poly ADP ribose (PAR) during DNA repair (Kumbhar et al. 2021). Consistent with its effect on PD-L1, niraparib significantly increased the expression of KDM5A in cervical cancer cells (Fig. 3a, b). As KDM5A is mainly localized in the nucleus, we examined the expression of KDM5A in the nucleus of cervical cancer cells treated with niraparib (Fig. 3c, d). ChIP assays showed that KDM5A bound to the *Pten* promoter region in HeLa cells in the presence of niraparib (approximately 2000 bp from the transcription start site) (Fig. 3e, f).

To further determine the inhibitory effect of KDM5A on PTEN, cervical cancer cells were transfected with siRNA to knock down KDM5A. As shown in Fig.3g, KDM5A knockdown in SiHa and HeLa cells significantly increased PTEN mRNA levels. Along with the knockdown of KDM5A, the expression level of PTEN increased, while the PD-L1 expression significantly decreased (Fig. 3h). To demonstrate the effect of niraparib on PD-L1 relied on KDM5A regulation of PTEN, we tested the efficacy of niraparib in KDM5A-knockdown cervical cancer cells. Neither the reduction in PTEN expression nor the expansion of PD-L1 abundance was observed in cervical cancer cells treated with the niraparib in the KDM5A knockdown cell lines (Fig. 3i). In summary, niraparib increased KDM5A expression in cervical cancer cells, which activated the PI3K-AKT-S6K1 signaling cascade and promoted PD-L1 accumulation.

.

**Fig. 4:**
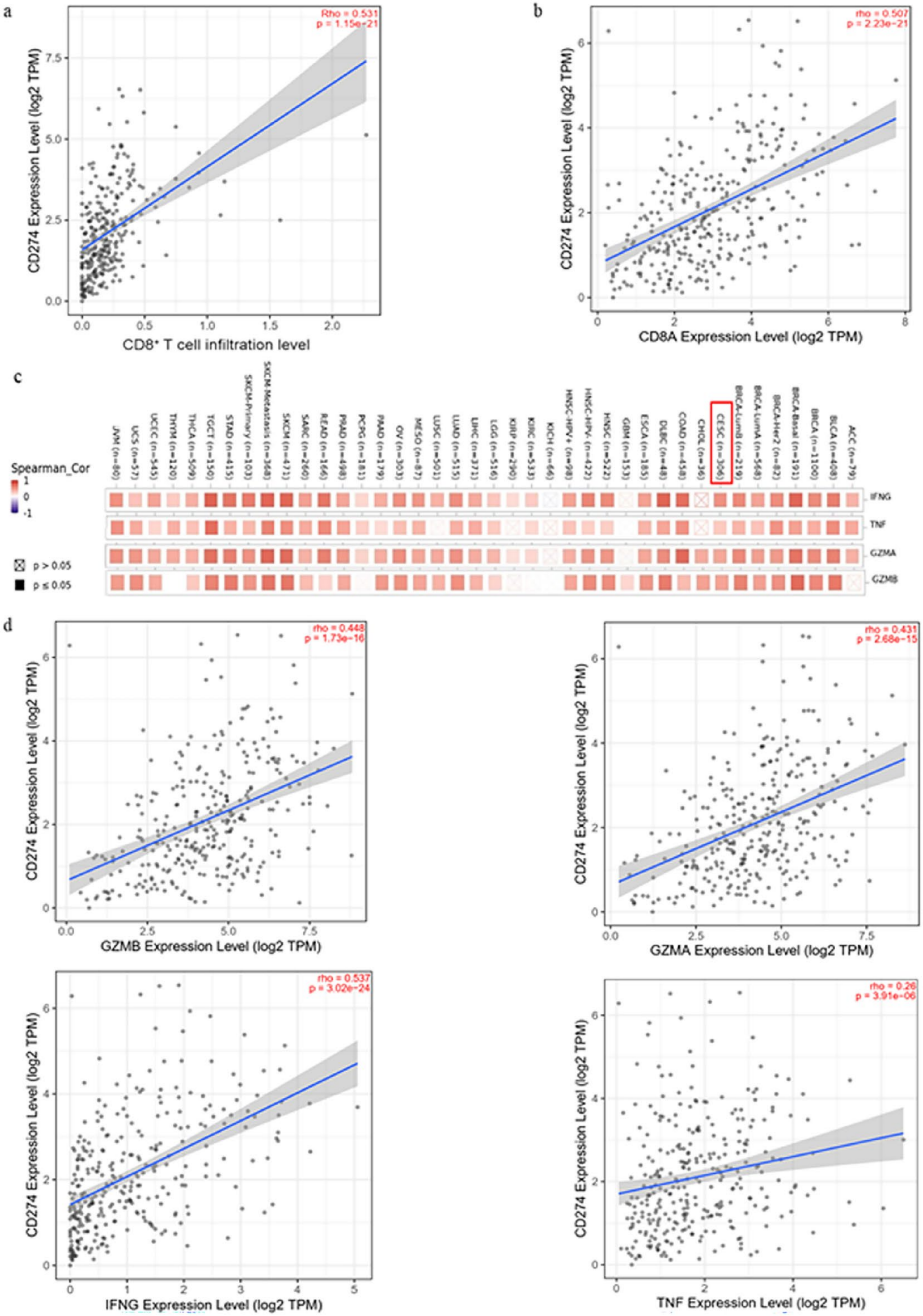
Relationship between PD-L1 and immune effector molecules. **a**,**b** The effect of PD-L1 on CD8^+^ T cell infiltration and CD8A expression in cervical cancer tissues, analyzed by TIMER2.0 database; **c** the correlation between PD-L1 and the expressions of immune effector molecules GZMA, GZMB, TNF, and IFNG in pancancer tissues was analyzed online by using TIMER2.0 database. **d** Online analysis of the correlation between PD-L1 and immune effector molecules GZMA, GZMB, TNF, and IFNG expression in cervical cancer tissues

### PD-L1 expression is positively correlated with immune effector molecules

The efficacy of immune checkpoint blockade is closely related to the infiltration of immune cells and expression of immune effector molecules in tumor tissues. We analyzed the correlation between PD-L1 and immune cells, as well as immune effector molecules, in cervical cancer tissues using the online TIMER2.0 database. The results showed that PD-L1 expression in cervical cancer was positively correlated with the infiltration of CD8^+^ T cells and the expression of CD8A (Fig. 4a, b). Similarly, there was a positive correlation between PD-L1 and immune effector molecules, such as TNF-α, IFN-γ, granzyme A, and granzyme B (Fig. 4c, d), which have been implicated in improving the immunosuppressive microenvironment.

### Niraparib enhances antitumor effects with PD‑L1 blockade in vivo

**Fig. 5:**
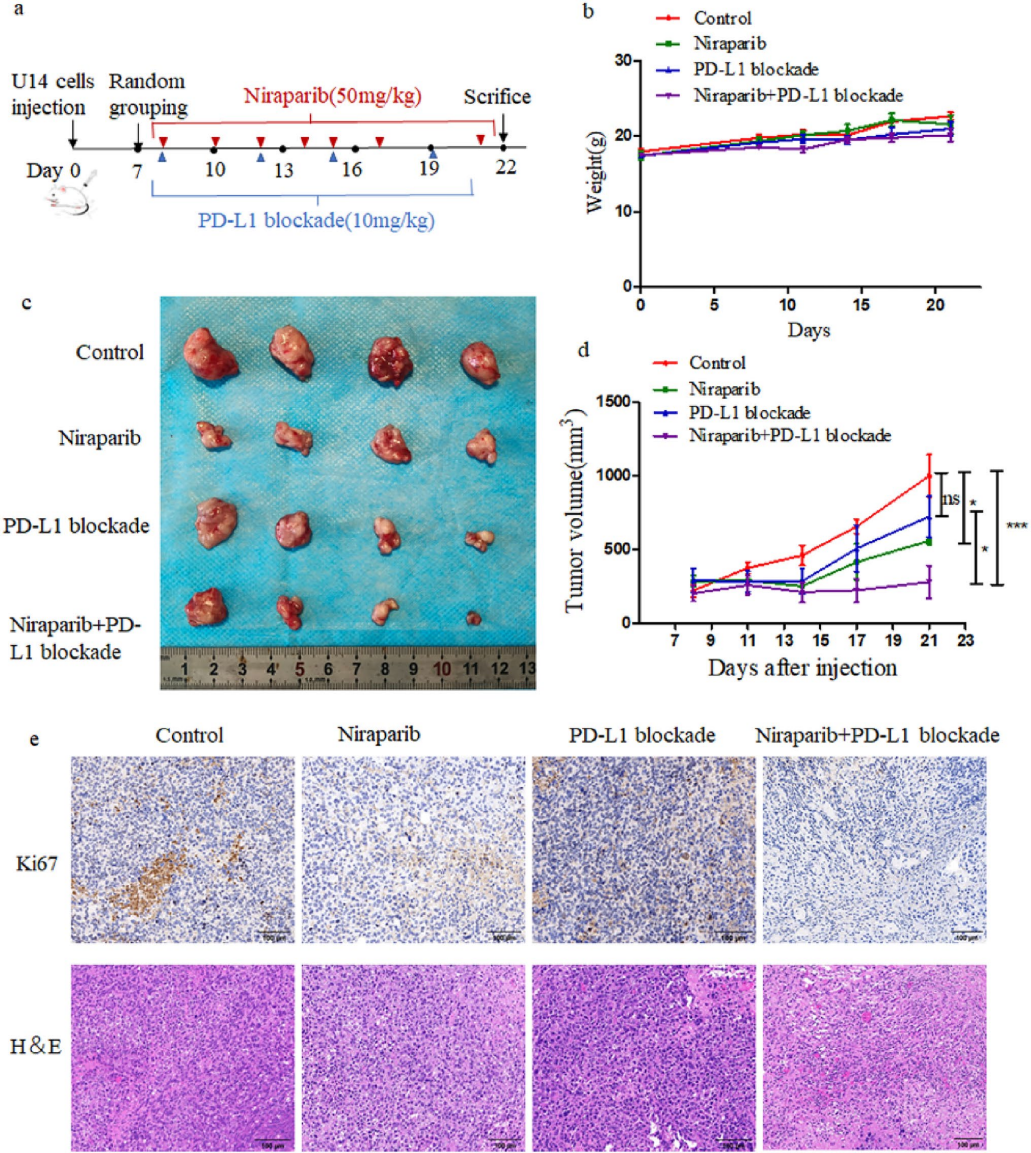
Niraparib has a synergistic effect with PD‑L1 blockade in vivo. **a** Schematic diagram of in vivo experiment design. **b** Weight curve of immunocompetent C57BL/6 mice treated with control, niraparib, PD-L1 blockade, and the combination for day 21 (*n* = 4). **c** Tumor volumes of four treatment groups with control (PBS, 100 μl), niraparib (50 mg/kg, 4 time per week), PD-L1 blockade (10 mg/kg, twice of the week), and niraparib with PD-L1 blockade. **d** Tumor growth curve of immunocompetent C57BL/6 models treated with control, Niraparib, PD-L1 blockade, and the combination for day 21 (*n* = 4). **e** Representative H&E staining and immunohistochemical analysis of Ki67 in tumors sections from different treated groups. Scale bar: 100 μm. Data were presented as the mean ± SD. Results were analysed by SPSS 25.0 software. *** *P* < 0.001,* *P* < 0.05, ns, not significant

Based on the effect of niraparib on cervical cancer cells in vitro, we sought to determine whether niraparib could further enhance the therapeutic effect of PD-L1 blockade in vivo. We treated immunocompetent U14 syngeneic mouse model with niraparib and/or PD-L1 blockade (Fig. 5a). There was no obvious change in the weight curve of mice, which indicated that the combination of niraparib and PD-L1 blockade had no more adverse effects than ICB alone (Fig. 5b). PD-L1 blockade monotherapy exerted moderate antitumor effect in the U14 tumor model, and when combined with niraparib caused a significant decline in tumor growth (Fig. 5c, d). Compared with the PD-L1 blockade monotherapy group, the tumor tissue structure was loosened and the proliferation index Ki67 was significantly reduced in the combined treatment group (Fig. 5e).

### Combination therapy with niraparib and PD-L1 blockade strengthens antitumor immunity in vivo

**Fig. 6:**
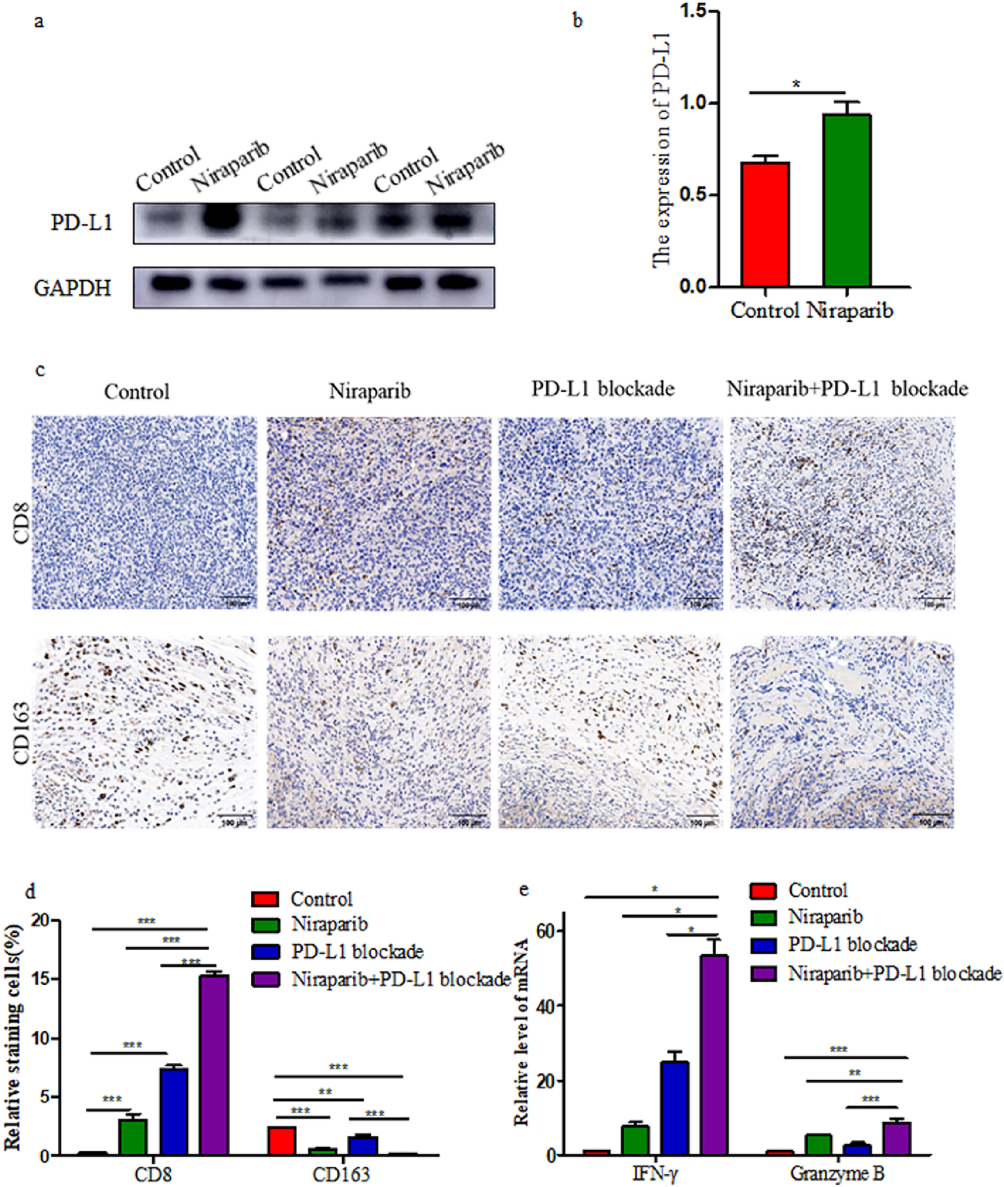
Combination therapy with niraparib and PD-L1 blockade improves antitumor immunity. **a**,**b** Immunoblotting analysis of PD-L1 expression in subcutaneous transplanted tumors treated with niraparib or PBS (*n* = 3). **c** Representative images of immunohistochemistry (IHC) staining of CD8^+^ T cells and CD163^+^ M2 TAMs in subcutaneous transplanted tumors after the indicated treatments. Scale bar: 100 μm. **d** Quantification of CD8^+^ T cells and CD163^+^ M2 TAMs with different treatment groups. **e** Relative expression of IFN-γ and granzyme B mRNA level from resected tumors with different treatments at day 21. Representative results were shown from three mice per group. Data were presented as the mean ± SD. Results were analysed by SPSS 25.0 software. *** *P* < 0.001, ** *P* < 0.01, * *P* < 0.05

We further evaluated how niraparib and PD-L1 blockade combination treatment affected the tumor microenvironment in a murine cervical cancer model. Following niraparib treatment, we observed a significant enhancement in PD-L1 expression in vivo using immunoblotting (Fig. 6a, b). We measured CD8^+^ T cells in subcutaneously transplanted tumors by immunohistochemistry (Fig. 6c). The quantity of intratumoral CD8^+^ T cells was significantly increased by the combination treatment (Fig. 6d). In addition, we detected a significant reduction of M2 tumor-associated macrophages (TAMs) in the combination therapy group compared to PD-L1 blockade and control groups. M2 TAMs (CD163^+^) were significantly reduced in niraparib group compared to the control group (Fig. 6c, d). Further analysis suggested that both niraparib and PD-L1 blockade caused appreciable changes in the mRNA expression of IFN-γ and granzyme B, but the combined treatment increased IFN-γ and granzyme B mRNA levels more significantly than the single treatment (Fig. 6e). Therefore, combination therapy with niraparib and PD-L1 blockade significantly enhanced antitumor immunity in cervical cancer model.

## Discussion

With the development of targeted therapies, ICBs and PARP inhibitors have provided new ideas for cancer treatment. Although ICB monotherapy has made inspiring breakthroughs in a variety of solid tumors, the development of drug resistance often remains a major problem (Ruiz de Galarreta et al. 2019). PARP inhibitors activate intrinsic immune pathways within tumor cells regardless of BRCA status, which lays the foundation for the combination of PARPi with ICB (Wang et al. 2019). In addition, impaired DNA damage repair induced by PARPi may increase tumor neoantigens, which may enhance the efficacy of ICB (Wu et al. 2021). However, there is limited evidence regarding the efficacy of combined therapy with PARP inhibitors and ICB in cervical cancer, and the potential mechanism warrants further study. Our study showed that niraparib could regulate the tumor immune microenvironment in cervical cancer, and when combined with PD-L1 monoclonal antibody in vivo, the inhibitory effect was significantly better than either monotherapy, which provided a solid theoretical basis for the treatment of metastatic or recurrent cervical cancer with PARPi combined with PD-L1 blockade.

Although PD-L1 is an imperfect biomarker owing to detection methods, non-standardized scoring systems, and tumor types, many studies have shown that high expression of PD-L1 is positively correlated with the PD-1/PD-L1 blockade response rate or OS (Wu et al. 2021; Rui et al. 2019; Ferris et al. 2016; Aguilar et al. 2019). The expression level of PD-L1 in tumor cells can be regulated by various signaling pathways, such as PI3K-AKT-mTOR, NF-κB and JAK-STAT (Yi et al. 2021). PARP inhibitors may upregulate PD-L1 expression on the surface of tumor cells through multiple pathways. In a mouse model of high-grade plasmacytoid ovarian cancer driven by concomitant deletion of p53, Brca1 and c-Myc overexpression, olaparib induced an increase in PD-L1 expression in tumor cells by activating the interferon gene stimulating factor (STING) pathway (Ding et al. 2018). PARP inhibitors can also inactivate GSK3β to increase PD-L1 expression in tumor cells, and combination with PD-L1 inhibitors can enhance the antitumor efficacy of PARP inhibitors (Jiao et al. 2017). We found that niraparib activated the PI3K-AKT-S6K1 signaling pathway in cervical cancer cells. Although it has been reported that gene changes in the PI3K/AKT pathway are related to immunotherapy and PARPi resistance (Peng et al. 2016; Borcoman et al. 2019; Han et al. 2019), emerging evidence indicates that activation of the PI3K/AKT pathway might be a good target for immunotherapy (Nusrat et al. 2019; Huang et al. 2021).

KDM5A and other KDM5 family proteases are associated with transcription and DNA damage response, in which KDM5A has a unique PAR binding region and PARPi can block the KDM5A-PAR interactions (Kim et al. 2019; Kumbhar et al. 2021). Our findings suggested that niraparib increased the expression of KDM5A in cervical cancer cells and promoted its binding to the *Pten* promoter region, resulting in reduced transcription and translation of *Pten*. The decrease of PTEN expression activated PI3K-AKT-S6K1 pathway and increased the expression of PD-L1, which was consistent with the conclusion previously reported that the deletion of PTEN causes an increase in PD-L1 expression in tumor cells (Song et al. 2013). The expression of PD-L1 was inhibited in the KDM5A knockdown cervical cancer cells and the inhibition could not be relieved by the addition of niraparib, indicating the importance of KDM5A in the regulation of PD-L1 expression by niraparib.

Cytotoxic T lymphocytes (CTLs) are the primary effector cells responsible for tumor killing (Yin et al. 2016), which mediated antitumor immunity forms the basis of immune elimination of cancer and underpins the efficacy of immune checkpoint blockade therapy (St Paul and Ohashi 2020). And studies have shown that PD-L1 expression is closely related to the infiltration of CD8^+^ T cells (Zhang et al. 2021). Consistent with previous results, we found that PD-L1 in cervical cancer tissue was positively correlated with the infiltration of CD8^+^ T cells and the expression of immune effector molecule through the analysis of biological information. Furthermore, we obtained relevant validation in vivo experiments. Our data demonstrated that niraparib could enhance the infiltration of CD8^+^ T cells, but decrease the abundance of M2 TAMs (CD163^+^) in the syngeneic mouse model. M2 TAM can not only be involved in the decreased infiltration of CD8^+^ T cells, but also secrete immunosuppressive molecules such as IL-10 and TGF-β in tumors tissues, which results in PD-1/PD-L1 blockade resistance (Pan et al. 2020; Dhupkar et al. 2018; Wu et al. 2022). IFN-γ activates and recruits T cells and plays an important role in anti-tumor immunity (Gao et al. 2018). Granzyme B represents the final signal under multiple immune regulatory pathways, reflecting the targeted killing ability of CTLs on tumor cells. It is a potential target for evaluating the effector function of tumor-specific CTLs (Trapani and Sutton 2003). We observed that compared with niraparib or PD-L1 blockade, the combination treatment could significantly increase the levels of IFN-γ and Granzyme B mRNA in tumor tissues, suggesting that the combination of niraparib and PD-L1 blockade therapy could reinforce the cytotoxic effector function of CTL, activate IFN-γ signaling pathway, and further strengthen antitumor immunity. Thus, these data indicate that niraparib enhances antitumor immune responses, and the combination of niraparib and PD-L1 blockade appears to be more effective than any single treatment.

## Conclusion

In conclusion, our study demonstrated that niraparib enhanced the efficacy of PD-L1 blockade in cervical cancer, offering a potential treatment strategy for advanced or recurrent cervical cancer. We verified the regulatory effects of niraparib on the immune microenvironment of cervical cancer. The efficacy of ICB in combination with PARPi in cervical cancer requires further confirmation in clinical trials.

## Data Availability

The datasets generated and/or analyzed during the current study are available in TIMER 2.0 (http://timer.comp-genomics.org/).
